# The flashy escape: support for dynamic flash coloration as anti-predator defence

**DOI:** 10.1098/rsbl.2024.0303

**Published:** 2024-07-31

**Authors:** Sanni Silvasti, Darrell J. Kemp, Thomas E. White, Ossi Nokelainen, Janne Valkonen, Johanna Mappes

**Affiliations:** ^1^ School of Natural Sciences, Macquarie University, Sydney 2109, Australia; ^2^ Department of Biological and Environmental Science, University of Jyväskylä, Jyväskylä 40014, Finland; ^3^ School of Life and Environmental Sciences, The University of Sydney, Sydney 2106, Australia; ^4^ Janne Valkonen Research and Consulting, Vesanka 41940, Finland; ^5^ Organismal and Evolutionary Biology Research Programme, Faculty of Biological and Environmental Sciences, Helsinki University, Helsinki 00790, Finland

**Keywords:** dynamic colour flashing, avian predator, touchscreen, flash illusion

## Abstract

Dynamic flash coloration is a type of antipredator coloration where intermittently appearing colour patterns in moving animals misdirect predator attacks by obscuring the precise location and trajectory of the moving prey. Birds and butterflies with differing dorsoventral wing coloration or iridescent surface structures may potentially benefit from such effects. However, we lack an understanding of what makes for an effective dynamic flash colour design and how much it benefits the carrier. Here, we test the effect of colour flashing using small passerine birds preying upon colourful, moving, virtual ‘prey’ stimuli on a touchscreen. We show that at fast speeds, green-to-blue flashing colour patterns can reduce the likelihood of pecks hitting the target, induce greater error in targeting accuracy and increase the number of pecks at a stimulus relative to similarly coloured non-flashing targets. Our results support the idea that dynamic flash coloration can deflect predatory attacks at fast speeds, but the effect may be the opposite when moving slowly.

## 1. Introduction

Many animals employ protective colour patterns as the first line of defence against predators [1]. Cryptic colour patterns, for example, can be highly effective in defeating detection and/or visual classification by predators. Mechanisms that facilitate crypsis such as disruptive coloration, countershading and background matching generally function best when an individual can remain stationary. This is because vision is particularly sensitive to motion [2–4], rendering many animals vulnerable while moving [1]. In response, adaptive evolution has occasionally favoured prey colour patterns that appear conspicuous when stationary yet exploit limitations in their predators’ visual systems when in motion. This includes patterns comprising high-contrast stripes or other repeated elements [5–7] that elicit ‘flicker-fusion’ or ‘motion dazzle’ effects. Banded snake patterns [6–9], for example, afford protection by inducing motion blur or confusing a predator’s perception of speed and trajectory. Another, more recently recognized, class of motion-based visual defences are dynamic ‘flash colours’ [10–13]. These colours intermittently flash or flicker while an animal moves and are hypothesized to deflect attacks by predators by confusing the precise location and trajectory of the moving prey (i.e. analogous to flash-lag illusions in humans) [14–16]. Hypothesized examples include the iridescent wings of morpho butterflies, the glossy cuticle of bottle flies, the contrasting dorsoventral wing colours of oystercatchers and the shimmering mirror-like scales of some fishes [10–12,17,18].

The defensive potential of dynamic flash coloration has been tested only infrequently [10,11,17,19–22]. Murali *et al*.’s [10,17,19] efforts are the most extensive to date and have used humans ‘preying upon’ stimuli on touchscreens to identify several parameters that contribute to the effectiveness of defensive flash colours. Namely, the size and flash-rate of a stimulus, and its speed and trajectory of movement [10,19]. Prior to these efforts, Palleroni *et al*. [21] tested dynamic flash colorations in feral pigeons (*Columba livia*). The pigeons evade peregrine falcon (*Falco peregrinus*) attacks by rotating in flight and rolling out of the falcon’s flight path while displaying differentially coloured rump and lower abdomen feathers sequentially at a fast pace [21]. They found that white-rumped pigeons are significantly more likely to survive attacks by the falcons compared with a phenotype with less contrasting rump feather coloration. Pike [11] tested the effect of touchscreen-simulated green-to-blue flashing iridescence of a bottle fly using Japanese quail (*Coturnix japonica*) as predators [11]. He found that, when compared with non-flashing stimuli, flashing induced greater targeting error and increased the number of attempts required to successfully peck the stimulus. Recently, Rao *et al*. [20] showed that interference coloration flashing in the wings of tephritid flies interrupts the visual tracking ability of predatory jumping spiders [20], and Henríquez-Piskulich *et al*. [22] showed that glossiness in a moving prey item can negatively affect the ability of praying mantids to track and accurately attack artificial prey [22].

Here, we primarily expand on Pike’s [11] research on the functional purpose of dynamic flashing by testing the efficacy of intermittently displayed colours in moving prey as an antipredator defence against a wild omnivorous passerine. Our study tests the relationship between different stimulus speeds on the effect of dynamic flashing both in colour and luminance. We used a touchscreen-based behavioural assay with a prey stimulus that simulates a flying animal with contrasting dorsoventral wing coloration and tested how this type of dynamic colour flashing works at different speeds of prey movement. Although animals can use colour flashing in signalling while not moving around, in this experiment, we are specifically interested in how dynamically flashing colours affect tracking and catchability moving prey. We tested stimuli of three different colours (monochromatic green, light green and blue) and two mixed-colour forms in which each side of the stimulus was differently coloured (alternating green-to-light green or green-to-blue) to test whether intermitted displaying of different colour and/or brightness affects the bird’s ability to catch the artificial prey. We predicted that colour flashing (i.e. display of alternating colours) should reduce catching success, decrease the likelihood of pecks hitting a stimulus, cause birds to have greater error distance in pecks towards targets and cause them to expend a greater number of pecks when attempting to catch the stimulus.

## 2. Methods

### (a) Experimental design

We used wild Eurasian blue tits (*Cyanistes caeruleus*, *n* = 20) caught from the Konnevesi Field station area (62.6165 N, 26.3457 E), Central Finland, which we housed in individual aviaries with food and water provided ad libitum when not being trained or tested. The birds were released to the catching site after experimenting.

The custom-made Touchscreen Operant Chamber (TOC) was an aviary consisting of a plywood (90 × 90 × 100 cm) with a touchscreen mounted into the rear wall and a feeder placed underneath. There was a camera and window opposite the touchscreen for observation. To create a touchscreen capable of responding to a bird’s beak and suitable for their visual system, we used a high-performance PC (Lenovo Legion T5 Ryzen 7 with 16 GB RTX 3070), and a high framerate gaming monitor (Asus ROG Swift PG329Q 32″ at 175 Hz) coupled with an infrared frame (G6 Integration Kit Touch Frame 6TP 32″, 250 fps maximum).

Prior to the behavioural assays, we trained birds to peck at dynamic (rotating, moving) virtual stimuli in three stages. First, we conditioned each individual to associate printed 2 cm dot stimuli with a food reward according to Silvasti *et al*. [23]. Second, birds learned to retrieve food from the remotely controlled feeder by pecking a dot situated on a grey cardboard background, after which the food reward was released. Finally, birds were habituated to peck at stimuli on a touchscreen in the TOC, introducing stimulus rotation and movement step by step. Training took 3–5 days and was considered completed when the birds had pecked each five rotating stimulus types on the touchscreen at least once when stationary and once when moving at varying speeds.

The stimuli are composed of two-dimensional, 20 mm flat disks which rotated horizontally around their own axis (like a paper-thin flipping coin) approximately 8 times per second. These were presented in five different colour treatments: green, light green, blue, green–light green (i.e. flash in luminance or ‘brightness’) and green–blue (i.e. flash in colour). The two dual-coloured stimuli were green on one side and blue or light green on the reverse side, and both contrasting sides appeared (flashed) with each rotation cycle. The rotating circular stimulus simulates laterally viewed wing beats of flying animals, such as birds, butterflies and others with relatively big and visible wings. The rotation frequency was inspired by a pierid butterfly wing flutter cycle recorded from a free-flying individual in glasshouse conditions.

Tests were conducted by presenting subjects with each of the five stimulus treatments sequentially in a randomized order. All stimulus types were first presented stationary and then at increasing speed in increments of *ca* 0.011 m s^−1^, and all stimulus types were presented in each speed round (see electronic supplementary material, table S1 on testing speeds and the duration that stimuli were available for pecking at each speed in electronic supplementary materials). The stimuli moved linearly across the mid-grey screen, either from left to right or vice versa, during which the birds had an opportunity to peck the stimulus (video on a bird pecking a stimulus in Dryad). Notably, pecking prey from a screen is not a natural foraging condition for the birds, and the stimulus speeds reflect this limitation. Even the fastest tested speed in this experiment is much slower than insect flying speeds generally (e.g. refer to flight speeds of some Lepidoptera in [24]).

The area in which a peck was registered as a ‘hit’ was defined by a circle of 20 mm centred on the stimulus, which did not vary with the change in stimulus size according to rotation. If not caught, the stimulus would move outside the bounds of the touchscreen, and if hit, the stimulus would disappear, and the bird would be rewarded from a remotely controlled feeder with a selection of mixed nuts and dried mealworms. If a bird failed at catching a stimulus, the same stimulus was spawned up to three times for the bird to attempt before being considered failed. Test concluded when the subject failed to catch any stimuli presented at a given speed. If the birds did not attempt to peck the offered stimulus, the same stimulus was repeated up to four times before it was considered refused.

Prior to testing, birds were food-deprived for 1 h to ensure motivation to forage. During experiments, we also gave the birds 30–40 min breaks when motivation appeared to lower.

### (b) Statistical analyses

We used generalized linear mixed models (GLMMs) to test the effect of stimulus colour treatment and speed on: (i) catch success (whether the subject managed to successfully peck the stimulus in a presentation), (ii) the probability of hitting the stimulus (given the total number of pecks in a presentation), (iii) targeting error distance (the average distance of missed pecks from the stimulus), and (iv) the number of pecks at the stimulus. In all models, we specified stimulus treatment, speed and the treatment × speed interaction as fixed effects, and subject ID as a random effect. We modelled catch success and peck probability of hitting stimulus using a binomial distribution, targeting error using a gamma distribution and the number of pecks using Poisson distribution, with a logit-link function in binomial distribution and log link in gamma and Poisson distribution. On top of the GLMMs described here, we ran similar analyses for the outcome of the first peck at a stimulus and the number of pecks before a hit (outlined in the electronic supplementary material, section ‘Additional analyses’ and electronic supplementary material, table S3).

We analysed planned contrasts (i.e. a *priori* contrasts informed by theory that maintain higher statistical power than pairwise post hoc tests [25]) to see how different stimulus types fared in 0 speed, mid-speed (4.5 cm s^−1^) and fast speed (9 cm s^−1^). The contrasts were analysed using the model outcomes of the four GLMM (i)–(iv) described above and were designed to test the effect of colour and luminance flashing. The tested contrasts were (1) green versus blue as a test for the effect of colour, (2) green versus light green as a test for the effect of luminance, (3) green and blue versus green-to-blue alternating stimulus as a test for the effect of colour flashing and (4) green and light green versus green-to-light green alternating stimulus as a test for the effect of luminance flashing. The colour and luminance flashing stimuli were contrasted to the mean of the two colours consisting of the flashing stimuli. The results are explained in the electronic supplementary material, section ‘Additional analyses’ and the electronic supplementary material, table S2.

We visually affirmed GLMM assumptions and used the 'lme4’ package (v. 1.1-34; [26]) for R (v. 2023.06.2; [27]) to estimate models, and the ‘emmeans’ package (v. 1.10.0; [28]) to analyse planned contrasts.

## 3. Results

**Figure 1.**
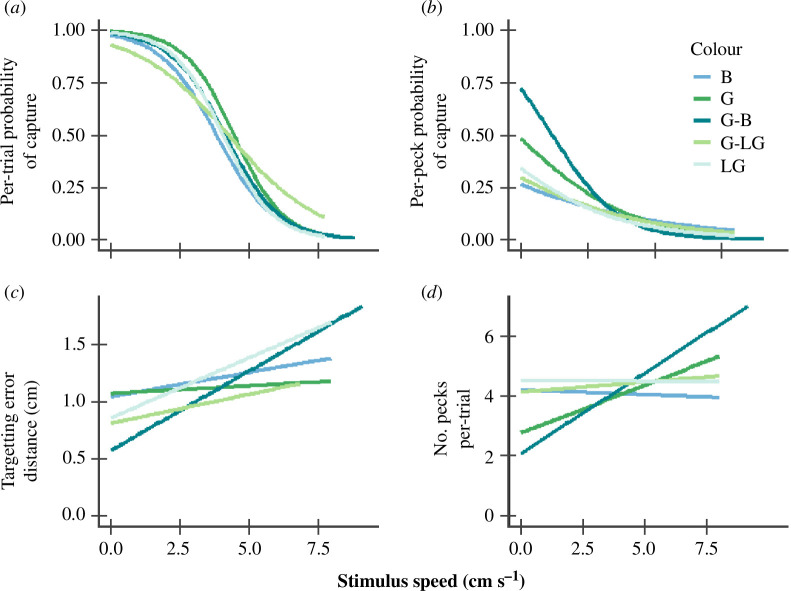
The effect of stimulus colour and speed upon: (*a*) a subject’s probability of successfully capturing (i.e. hitting) a stimulus during each trial, (*b*) the likelihood of any single peck hitting the stimulus, (*c*) the mean distance of missed pecks from the stimulus edge and (*d*) the number of pecks expended per-trial. Colour abbreviations are B = blue, G = green, G-B = green–blue, G-LG = green–light green and LG = light green.

Overall catch success (i.e. across all speeds) did not differ between the stimulus types (figure 1*a*, table 1), but there were differences in the amount of effort required to capture them. Per-peck probability of hitting the target stimulus varied between stimulus types and different test speeds; when the stimuli did not move, green-to-blue flashing stimulus type had the highest per-peck probability of being hit, while blue stimulus had the lowest per-peck probability of being hit (figure 1*b*, table 1). As stimulus speed increased, the probability of pecks hitting a stimulus decreased most rapidly for the green–blue stimulus (figure 1*b*, table 1), and pecks were significantly less likely to hit the green-to-blue flashing stimulus in fast speed when contrasted against green and blue non-flashing stimuli (electronic supplementary material, table S2). Targeting error also differed between stimulus types (figure 1*c*, table 1). With increasing speeds, green–blue flashing and light green stimulus types had larger missed peck distances compared with other stimuli. This is supported by planned contrast where light green stimulus had a significantly larger targeting error compared with green, and green-to-blue flashing had a significantly larger targeting error compared with the average targeting error of green and blue non-flashing stimuli (electronic supplementary material, table S2). The number of pecks at stimuli varied, with green-to-blue flashing stimulus having the most pecks with increasing speeds followed by the green stimulus type (figure 1*d*, table 1).

**Table 1.** Results for the three generalized linear mixed models, where colour blue is the intercept on catch success, likelihood of pecks hitting target, missed peck error distance and number of pecks. Stimulus colours are abbreviated as: G = green, G-B = green–blue, G-LG = green–light green, LG = light green.

	estimate	standard error	*z* value	*Pr*(>|*z*|)
catch success				
intercept	4.044	0.718	5.630	**1.80 × 10^−8^**
G	1.373	1.185	1.159	0.247
G-B	0.547	1.036	0.528	0.598
G-LG	−1.178	0.856	−1.377	0.168
LG	0.828	1.082	0.766	0.444
speed	−1.187	0.211	−5.635	**1.75 × 10^−8^**
G:speed	−0.179	0.324	−0.554	0.580
G-B:speed	−0.055	0.295	−0.186	0.853
G-LG:speed	0.424	0.250	1.697	0.090
LG:speed	−0.151	0.310	−0.486	0.627
likelihood of pecks hitting stimuli				
intercept	−0.924	0.220	−4.204	**2.62 × 10^−8^**
G	0.970	0.301	3.219	**0.001**
G-B	1.957	0.353	5.541	**3.01 × 10^−8^**
G-LG	0.246	0.292	0.843	0.399
LG	0.450	0.298	1.509	0.131
speed	−0.345	0.079	−4.396	**1.10 × 10^−5^**
G:speed	−0.217	0.119	−1.830	0.067
G-B:speed	−0.579	0.136	−4.257	**2.07 × 10^−5^**
G-LG:speed	−0.058	0.115	−0.502	0.616
LG:speed	−0.207	0.126	−1.641	0.101
missed peck error distance				
intercept	−0.0251	0.0948	−0.265	0.791
G	−0.0928	0.1170	−0.792	0.428
G-B	−0.3373	0.1305	−2.584	**0.010**
G-LG	−0.2339	0.1093	−2.140	**0.032**
LG	−0.3068	0.1069	−2.870	**0.004**
speed	0.0794	0.0208	3.808	**0.000**
G:speed	−0.0115	0.0338	−0.340	0.734
G-B : speed	0.0697	0.0348	2.004	**0.045**
G-LG:speed	0.0056	0.0334	0.167	0.867
LG:speed	0.0835	0.0331	2.521	**0.012**
number of pecks				
intercept	1.3570	0.1100	12.337	**2 × 10^−16^**
G	−0.4097	0.1192	−3.436	**0.000**
G-B	−0.6541	0.1225	−5.339	**9.36 × 10^−08^**
G-LG	−0.0318	0.1107	−0.288	0.774
LG	0.0720	0.1085	0.664	0.507
speed	−0.0063	0.0261	−0.242	0.809
G:speed	0.1155	0.0370	3.122	**0.002**
G-B:speed	0.1969	0.0361	5.460	**4.77 × 10^−08^**
G-LG:speed	0.0353	0.0355	0.994	0.320
LG:speed	0.0127	0.0356	0.356	0.722

## 4. Discussion

The potential for temporally dynamic colour patterns to act as antipredator defences while an animal moves has empirical support [10–13,17,19–21], but the breadth of such effects is still not fully understood. Here, we have used a touchscreen-based assay to demonstrate that coloration and colour flashing can influence how wild-caught birds localize moving dynamic visual targets. The most salient finding consisted of an interaction between stimulus speed and colour flashing. Whereas the green-to-blue stimulus was relatively easiest to catch when stationary and at low speed (judged according to both per-peck probability of hit and targeting error; figure 1*b,c*, electronic supplementary material, table S2), capture ability decreased more steeply with increasing speed. At the highest speed, green-to-blue flashing stimulus had significantly lower per-peck probability of being hit compared with non-flashing green and blue stimuli (electronic supplementary material, table S2), elicited the largest targeting error compared with all stimulus types (figure 1*c*) and required the highest number of pecks for successful capture (figure 1*d*, electronic supplementary material, table S3).

Overall capture success across all speeds did not vary between stimulus types (figure 1*a*, table 1), most likely because test subjects had the opportunity to repeatedly peck at a stimulus as it traversed the touchscreen. This differs from natural predator–prey interactions, where a single failed predatory attack may often be enough to elicit escape behaviour. However, there were differences in likelihood of pecks hitting a target across stimuli, targeting error distances of missed pecks and number of pecks per stimulus type. When non-moving, the green–blue and green treatments were especially easy to hit, whereas the blue stimuli were particularly difficult (figure 1*b*, table 1, electronic supplementary material, table S2). With fast speeds, pecks were significantly less likely to hit the green–blue stimulus compared with the non-flashing green and blue stimuli on average (figure 1*b*, table 1, electronic supplementary material, table S2). The number of pecks also increases notably in the green–blue flashing and green stimulus types with fast speeds (figure 1*d*). When considered in concert with the large targeting error distances (figure 1*c*), we conclude that birds invested more effort in catching moving green–blue stimuli in fast speeds compared with the other stimulus types. The birds appeared highly motivated to catch the green–blue stimulus type as they kept trying to catch it despite it being difficult. Our findings are analogous to Pike’s [11] result with Japanese quail having larger targeting errors and more pecks at green-to-blue flashing stimuli compared with non-flashing stimulus types.

Interestingly, the luminance flashing green-to-light green stimulus type did not seem to cause the birds more difficulties compared with other stimuli; perhaps the alternating colours did not have large enough luminance contrast to induce dynamic flash effects. Meanwhile, the light green stimulus caused birds to have large targeting errors in fast speed (figure 1*b*, table 1, electronic supplementary material, table S2), and the blue stimulus had the lowest per-peck probability of being hit while non-moving (table 1, electronic supplementary material, table S2). Several studies considering visual defences in motion have also shown that other than the hypothesized stimulus types can sometimes provide protection. For example, a mid-grey stimulus type often provides similar defence as motion dazzle and dynamic flash colorations against human predators on touchscreen-based tests [5,10,29,30]. We hypothesize that differently coloured targets may vary in catching difficulty in different situations, including a chance that signalling background and ambient light on top of speed of movement may affect how easily a moving prey is caught.

The stimulus rotation frequency and faster movement speeds that were used in this experiment may also affect how easily stimuli are hit. The rotation frequency used here was inspired by butterfly flight recorded in controlled conditions. However, if feeling threatened, the butterfly flutter cycle would likely become much faster. Birds will also face faster moving prey in nature than could be tested here, as pecking the touchscreen posed challenges. Murali [10] tested artificial prey stimuli on a touchscreen with humans using flash frequencies of 5, 10 and 15 Hz with several stimulus speeds [10]. He found that the highest flashing frequency caused larger targeting errors compared with the slower flashing stimuli, but much like in our experiment, there was no difference in the overall catching successes between the tested stimuli.

It is important to note that natural prey animal movement is generally erratic and unpredictable [31]. This so-called protean movement strategy serves an antipredator function [32], and empirical work has shown it to improve the efficacy of dynamic flash coloration as it renders prey even more difficult to locate [19]. The additional difficulty imparted by naturalistic movement may broaden the gamut of potential colour pattern and flash designs for use as antipredator defences. Notably, birds found the task of pecking rotating stimuli difficult, leading us to adjust the hitbox to a constant circle even though stimulus area varied with rotation. This raises the standing question of whether, for example, dynamic shape changes associated with wing fluttering in insects with opaque and/or colourful wings impair a predators ability to track the location and trajectory of prey [12].

In this work, we found that dynamic flash coloration in fast moving prey can improve the chances of escaping capture by predators further supporting the idea that flashing can hamper the localization of moving objects. However, much like in other experiments on anti-predator coloration that function in motion [5,10,29,30], dynamic colour flashing does not grant equal protection in all movement speeds nor is it unique in the sense that similar protection may sometimes be achieved with uniform coloration. The attack deflecting effect is likely caused by flash illusions that arise when the visual system’s predictive motion processing is impaired by unexpected features in the viewed scene [16]. The exact attributes of effective dynamic flash coloration for specific visual systems remain unresolved, as does the effect of variation in visual environments where the patterns are displayed. The future likely encompasses exciting new revelations regarding dynamic flash colour designs and their interactions with target visual systems and environments. We may also expect to acknowledge a variety of previously unrecognized dynamic flash effects in nature under different signalling contexts.

## Data Availability

The data presented and analysed in this manuscript, and a video of a bird during a trial, are available in the Dryad Digital Repository [33]. Supplementary material is available online [34].
